# Microbiome therapeutic PMC72 through reverse translational research in gout

**DOI:** 10.71150/jm.2501002

**Published:** 2025-05-27

**Authors:** Mohammed Solayman Hossain, Hoonhee Seo, Kyung-Ann Lee, Asad ul-Haq, Sukyung Kim, Sujin Jo, Md Abdur Rahim, Hanieh Tajdozian, Fatemeh Ghorbanian, Youjin Yoon, Indrajeet Barman, Md Sarower Hossen Shuvo, Hyun-Sook Kim, Ho-Yeon Song

**Affiliations:** 1 Department of Microbiology and Immunology, School of Medicine, Soonchunhyang University, Cheonan 31151, Republic of Korea; 2 Human Microbiome Medical Research Center, School of Medicine, Soonchunhyang University, Asan 31538, Republic of Korea; 3 Division of Rheumatology, Department of Internal Medicine, Soonchunhyang University Seoul Hospital, Seoul 04401, Republic of Korea

**Keywords:** microbiome therapeutics, gout, hyperuricemia, *Bifidobacterium*, reverse translational research

## Abstract

Gout is an inflammatory arthritis resulting from the deposition of monosodium urate crystals. Urate-lowering therapies for gout have limitations, including side effects and limited efficacy, highlighting the need for novel therapeutic approaches to improve patient outcomes. In this context, our research team conducted a microbiome analysis of fecal samples from healthy individuals and gout patients, identifying *Bifidobacterium* as a key biomarker. Subsequently, we isolated and identified this strain, *B. longum* PMC72, and demonstrated its efficacy in a gout mouse model. In potassium oxonate (PO)-induced hyperuricemia mice, PMC72 significantly alleviated nausea, gait disturbances, ankle inflammation, and improved renal health. These effects were associated with marked reductions in oxidative stress markers, including serum uric acid, blood urea nitrogen, hepatic xanthine oxidase, and malondialdehyde (MDA) levels in serum, liver, and joint samples, as well as the downregulation of inflammation and uric acid transport-related gene expression in kidney samples. These benefits were comparable to those treated with Febuxostat, a standard urate-lowering therapy for gout. Furthermore, gut microbiome analysis revealed that PMC72 restored dysbiosis induced by hyperuricemia, contrasting with the reduced microbial diversity observed with febuxostat alone, and showed a complete recovery to eubiosis when combined with Febuxostat. These findings position PMC72 as a promising microbial therapeutic candidate for gout management, demonstrating significant development potential and serving as a benchmark for reverse translational microbiome-based therapeutic research.

## Introduction

Gout, an inflammatory arthritis resulting from the deposition of monosodium urate crystals (MSU) in joints, has become an escalating global health concern with a substantial increase in prevalence in recent years (Sivera et al., 2022). Over the last two decades, there has been a noteworthy 63.44% surge in global gout incidence, accompanied by a parallel increase of 51.12% in global years lived with disability (He et al., 2023). As of 2023, the global tally of reported gout cases reached a significant milestone, totaling 100 million (He et al., 2023). Elevated serum urate level (hyperuricemia) is the key risk factor for developing gout. Precipitation of MSU crystals typically occurs when their concentration in a solution exceeds 404 µmol/L (6.8 mg/dL), particularly under conditions like low temperatures and acidity (Oliveira and Burini, 2012). Furthermore, gout is often linked to comorbid conditions such as hypertension, obesity, cardiovascular disease, diabetes, dyslipidemia, chronic kidney disease, and kidney stones. These comorbidities can complicate gout management and significantly increase the risk of premature mortality (Gliozzi et al., 2016). Observational studies indicate that only 20% of patients with asymptomatic hyperuricemia and serum urate levels above 9 mg/dL develop gout within five years (Campion et al., 1987). Furthermore, the benefits of urate-lowering therapy do not outweigh the potential treatment risks. Therefore, prescribing medications to lower uric acid levels is not recommended for patients with asymptomatic hyperuricemia (FitzGerald et al., 2020). In patients with gout, urate-lowering agents such as febuxostat or allopurinol are key treatment strategies to reduce the frequency of gout attacks and prevent complications associated with the disease (Lee et al., 2023). However, due to the side effects of these medications and their insufficient efficacy in some patients, the development of safer and alternative therapies is essential for managing gout and hyperuricemia (Miyata et al., 2016). Given these challenges, exploring innovative approaches beyond traditional therapies has gained attention. In this context, microbiome-based therapy has emerged as a promising avenue, as its effectiveness has been well-documented in related fields (Tong et al., 2022).

The human microbiome includes all microbes and their associated metabolites in and on the human body (Walter and Ley, 2011). Microbes’ extensive role in human health holds excellent potential for use in disease management therapies. By interacting with the host, microbes can help prevent the onset of diseases, making them valuable for developing microbiome therapeutic development (Rahim et al., 2024; Thaiss and Elinav, 2017). For instance, *Akkermansia muciniphila* alleviates metabolic disorders, enhances the effectiveness of metformin in cancer treatment, and protects against atherosclerosis by reducing gut permeability and inflammation (Cuesta-Zuluaga et al., 2017; Li et al., 2016; Verdura et al., 2019). **Lactobacillus* johnsonii* offers protection against cancer (Cheema et al., 2016), while *Bifidobacterium longum* lessens the severity of Crohn’s disease and restores the mucus layer damaged by a high-fat diet (Joossens et al., 2012; Schroeder et al., 2018). Besides, studies conducted by Wu et al. (2021) and Kuo et al. (2021) revealed the urate-lowering effects and modulation of gut microbial composition by *L. fermentum* JL-3 (Wu et al., 2021), *L. reuteri* TSR332, and *L. fermentum* TSF331 in mouse models, respectively (Kuo et al., 2021). As knowledge about gut microbes expands, new opportunities for disease diagnosis, testing methods, and data collection techniques have emerged, offering fresh hope in therapeutic development (Yadav and Chauhan, 2022).

Considering the role of gut microbiota in human gout treatment, we previously conducted a study using clinical samples to examine the correlation between the gut microbiome and gout. Our prior research, as detailed in ul-Haq et al. (2022), conducted a meta-taxonomic analysis of stool samples and compared the gut microbiome of healthy controls with controlled and uncontrolled gout. In that study, we established a correlation between gut microbes and gout, identifying *Bifidobacterium* as a prominent biomarker in healthy controls. In contrast, *Prevotella* was more prevalent in individuals with uncontrolled gout. Identifying *Bifidobacterium* as a biomarker in this context provided a critical foundation for the subsequent reverse translational study. Reverse translational studies bridge the gap between clinical observations and experimental research by translating human-derived findings, such as microbial biomarkers, into targeted preclinical investigations (Gopalakrishnan et al., 2020). This approach allows for testing therapeutic interventions in controlled experimental models, ensuring that findings from human studies inform and refine potential treatments (Ghanem et al., 2023). Building upon our initial findings, the present study employs this reverse translational approach by exploring the therapeutic potential of *Bifidobacterium* through an in vivo investigation using a potassium oxonate-induced mice model of gout.

By unraveling the intricate interplay between PMC72 supplementation and gout-related markers, this study aims to contribute to the expanding knowledge of microbiome-based therapeutic strategies. Reducing uric acid levels and mitigating inflammation in vivo in mice would represent a significant breakthrough. Given the current challenges in managing gout, exploring probiotics as a viable and safer alternative holds great promise for revolutionizing treatment approaches and improving patient outcomes.

## Materials and Methods

### Isolation of biomarker from clinical sample

Therapeutic biomarker isolation commenced with collecting clinical samples of healthy individuals and hyperuricemia patients. 17 stool samples were collected from healthy participants at Soonchunhyang University Seoul Hospital. We used various techniques, optimizing media with various supplements, temperature, and oxygen requirements and isolating our targeted biomarker strain. Upon receiving, samples underwent serial dilution, with 1 g diluted to 1 ml of PBS solution, followed by subsequent dilutions of 10^-1^, 10^-2^, 10^-3^, 10^-4^, 10^-5^, and 10^-6^ before streaking on plates. Each sample was applied to BS agar plates (Kisan Bio, Korea) supplemented with 50 mg/L mupirocin, 1 g/L acetic acid, and BSM agar (Sigma, USA) to create a selective environment and incubated in an anaerobic chamber (Baker Ruskin, Canada) at 37°C for 48 h. After colony development, cultures were propagated into BS broth (Kisan Bio, Korea) following the same conditions and preserved at -80°C using 30% glycerol for further uses.

### 16S rRNA-based identification of isolated biomarker strains

Cultured strains were sent to a commercial sequencing company (Biofact, Korea) to identify the isolated biomarker strains, and initial identification was conducted using 16S rRNA gene sequencing technology. Briefly, after extracting DNA, PCR was performed using primers 27F (5'-AGAGTTTGA TCCTGGCTCAG-3') and 1492R (5'-GGTTACCTTGTTACGACTT-3') on a Hushrun PCR cycler (Biofact, Korea). The PCR product was purified and sequenced using an ABI PRISM 3730XL DNA analyzer (Applied Biosystems, USA) with a BigDye Terminator v3.1 Cycle Sequencing Kit (Thermo Fisher Scientific, USA). BLAST was used to compare sequences against the National Center for Biotechnology Information (NCBI) GenBank database.

### Whole-Genome Sequencing of probiotic strain

Whole-genome sequencing (WGS) analysis was conducted to determine the genome of the biomarker. The bacteria was introduced into BSM broth with an inoculum of 0.2%. Cells were harvested during the late exponential growth phase. Subsequently, genomic DNA (gDNA) extraction was performed using the QIAamp DNA Mini Kit from Qiagen after the cells were washed thrice with PBS. PacBio library preparation and whole-genome sequencing were conducted by ChunLab, Korea. PacBio sequencing data was processed using PacBio SMRT Analysis 2.3.0 with the HGAP2 protocol, and resulting contigs from the PacBio sequencing were circularized using Circlator 1.4.0 (Sanger Institute, UK). Prodigal 2.6.2 (Hyatt et al., 2010) was employed to predict protein-coding sequences (CDSs), which were subsequently classified based on their roles using orthologous groups (EggNOG; http://eggnogdb.embl.de). Gene identification for tRNAs (Lowe and Eddy, 1997) was performed using tRNAscan-SE 1.3.1, while rRNAs and other noncoding RNAs were identified via covariance model searches utilizing the Rfam 12.0 database (Nawrocki et al., 2015). The OrthoANIu algorithm-based Average Nucleotide Identity (ANI) calculator was utilized to compare prokaryotic genome sequences (Yoon et al., 2017).

### Biomarker strains preparation for animal model

Biomarker *B. longum* and *B. bifidum* strains were cultured in 30 ml of BS broth and incubated at 37°C in an anaerobic chamber for 24 h. Cultures were adjusted to OD 1.0 at 600 nm using a spectrophotometer (Hatch, USA). After that, centrifugation was performed at 4,000 rpm for 30 min using a centrifuge machine (Hanil Scientific, Korea), followed by washing with 0.85% NaCl solution to remove medium components. After that, the pellet was resuspended using 1 ml of 0.85% NaCl solution.

### Animal study design

This experiment investigated the effectiveness of Febuxostat and probiotics in mitigating the effects of potassium oxonate-induced gout in mice by analyzing changes in weight, joint health, and gut microbiota. Ten-week-old male BALB/c mice were acquired from DooYeol Biotech, Korea. The mice underwent a one-week acclimation period before the commencement of the experiments. After 1 week of environmental adjustment, mice were randomly divided into 6 groups (n = 5/group) named NC = Normal Control, DM = Disease Model induced by potassium oxonate, DM + Feb = DM treated with Febuxostat, DM + *B. longum* = *B. longum* treated group, DM + *B. bifidum* = *B. bifidum* treated group, DM + *B. longum* + Feb = Co-treatment of *B. longum* and Febuxostat in deceased model. Potassium oxonate was injected with 300 mg/kg and Febuxostat 5 mg/kg, and the concentration of biomarkers was 1 × 10^8^ CFU/ml. In preparing and injecting potassium oxonate (PO) to induce gout in mice, PO was suspended in 0.85% NaCl and filtered to ensure clarity and sterility. Febuxostat or probiotics were administered to the mice by oral gavage 1 h after the PO treatment for 10 days. The changes in the physical condition, such as overall health, body weight, ankle size, and walking pattern, of all the groups were noted daily during the experiment. The ankle size of the mice was measured using Vernier Calipers. After 10 days, all mice were sacrificed. Blood samples were collected, centrifuged, and stored at -80°C, while liver, large intestine, and kidney samples were prepared and stored accordingly for biochemical, Next Generation Sequencing (NGS), and histopathological analyses.

### Ankle size, sickness behaviour score, and walking pattern measurement

The diameter of mice’s ankles was a measure to assess gout pre- and post-treatment in the animals. The ankle circumference of all mice was measured before and after PO injection using a vernier caliper. The swelling ratio was calculated using the formula: Swelling ratio = (C_t_ − C_0_)/C_0_, where Ct represents the circumference of the ankle at various times, and C_0_ is the initial circumference at 0 h (Tang et al., 2017). The mice’s sickness behaviour score and walking pattern changes were calculated following Patil et al. (2021). Briefly, inflammation and dysfunction of mice were observed to measure the level of sickness and walking pattern index, respectively. The observed mice were classified into four grades based on specific criteria as of Grade 0–3, each with 2 points. Five well-trained volunteers marked mice based on external behaviour and morphology before sacrificing them. All of them were unaware of the treatment procedure. To assess sickness behaviour score and walking patterns, each volunteer separately scored each mouse, and the average score was calculated later. Scores ranged from 0 to 6, indicating the best to worst performance, equivalent to grades 0–3.

### Renal histopathological analysis

The removed mouse kidneys were placed in formalin and sent to KP&T, Korea, for histopathological analysis. The kidney tissues underwent initial fixation using 4% paraformaldehyde, followed by dehydration and subsequent embedding in paraffin. Thin sections of 5 µm were precisely cut, and these sections were then stained with hematoxylin and eosin (H&E). Images were obtained using the standard virtual tissue scan (Motic EasyScan Pro 6, China). The stained sections were meticulously examined under Microscope CX31 Upright, Olympus (Japan), to discern and analyze histopathological alterations at various magnifications.

### Biochemical assay

Blood and liver biochemical analyses were conducted to assess the levels of Serum Uric Acid (SUA), Blood urea nitrogen (BUN), and Xanthine oxidase (XOD). Commercial metabolism assay kits from Elabscience (USA) were employed for these analyses following manufacturer protocol. Briefly, 25 µl of collected blood samples were diluted using 0.85% NaCl solution and centrifuged at 2,000 rpm for 5 min. Afterward, the supernatant was transferred into a fresh tube, mixed with reagents 3 and 4, incubated at room temperature for 15 min, and finally, OD was measured using a microplate reader.

For XOD analysis, the liver sample was thoroughly homogenized by utilizing a Lysing Matrix B tube (MP Biomedicals, USA). Then, the sample was centrifuged at 3,000 rpm for 10 min, and the supernatant was collected. A standard curve was prepared along with the sample in all cases. Colorimetric assay reagent was added, mixed, and kept at room temperature for 15 min. Samples were dispensed in a 96-well plate to measure the OD values of each well at 690 nm and 550 nm using a microplate reader at 30-min intervals.

### MDA level analysis

The Malondialdehyde (MDA) level in the test animals’ serum, liver, and ankle joints was measured using a commercial metabolism assay kit (Elabscience, USA) following the manufacturer’s instructions. Initially, 20 mg of liver and ankle joint specimens were homogenized in 180 µl of PBS through mechanical lysis with a homogenizer (FastPrep-24 5G, MP Biomedicals, USA). After homogenization, the samples were centrifuged at 9,000 rpm for 10 min at 4°C to collect the supernatants. Following this, 100 µl of each sample (serum and supernatants of liver and ankle joint) was combined with 100 µl of clarificant reagent, 3 ml of acid application solution, and 1 ml of chromogenic application solution separately. Subsequently, the mixture was incubated at 100°C for 40 min, and then the optical density was measured at 532 nm using a spectrophotometer (Perkin Elmer, USA).

### Inflammatory gene expression analysis

Gene expression of mRNA from mice kidney samples was explored to evaluate the inflammation changes that happened before and after probiotic treatment. An RNeasy Mini kit (Qiagen, Germany) extracted RNA from kidney samples per the manufacturer’s guidelines. Subsequently, extracted RNA was quantified by Qubit Fluorometer (Invitrogen, USA) using a Qubit RNA Assay kit (Thermo Fisher, USA). Next, 1 μg of RNA was reverse transcribed to cDNA using the iScript cDNA synthesis kit (Bio-Rad, USA). Following this, RT-PCR was performed in a CFX96 Real-Time PCR Detection system (Bio-Rad, USA) with the following conditions: 95℃ for 30 s, followed by 40 cycles of 95℃ for 5 s and 60℃ for 30 s. The relative mRNA expressions were determined using the previously described method (Schmittgen et al., 2000).

### NGS-based analysis of mice gut sample

A 16S rRNA-based amplicon sequencing study of mice colon samples investigated the gut microbiome composition across six experimental groups treated with febuxostat and *Bifidobacterium* strains. Total DNA was extracted from each sample using a QIAamp DNA Mini Kit (Qiagen, Germany) following the manufacturer's instructions. DNA concentration was measured with a Qubit Fluorometer (Invitrogen, USA) and dsDNA HS Assay Kit (Thermo Fisher, USA), and its quality was assessed via agarose gel electrophoresis using a ChemiDoc imaging system (Bio-Rad, USA). The V4 hypervariable region of the 16S rRNA gene was amplified using universal primers specific to this region (Forward primer: TCGTCGGCAGCGTCAGATGTGTATAAGAGACAGCCTACGGGNGGCWGCAG; Reverse primer: GTCTCGTGGGCTCGGAGATGTGTATAAGAGACAGGACTACHVGGGTATCTAATCC). Agencourt AMPure XP beads (Beckman Coulter, USA) were used for PCR product purification after each amplification step. PCR product quantification was performed using a Qubit dsDNA HS Assay Kit. Per the manufacturer’s instructions, the metagenomic libraries were prepared using a Nextera XT DNA Library Prep Kit (Illumina, USA). After normalization, pooling, and mixing with 10% PhiX Control Library v3 (Illumina, USA), sequencing was conducted on the Illumina iSeq 100 platform (Illumina, USA).

The quantitative insights into microbial ecology (QIIME 2) software (version 2024.5) was used for sequence analysis (Bolyen et al., 2019). The DADA2 algorithm was applied to denoise the data, resolve Amplicon Sequence Variants (ASVs), and perform quality filtering, removing chimeric sequences and correcting sequencing errors (Callahan et al., 2016). Taxonomic classification was performed using the SILVA 16S rRNA reference database, and microbial composition was analyzed from phylum to genus levels (Gao et al., 2017). To assess microbial diversity, α-diversity indices such as Shannon and Simpson were calculated to measure species richness and evenness. β-Diversity was evaluated based on Jansen-Shannon diversity, and principal coordinates analysis (PCoA) was used for visualization (Chao and Shen, 2003; Lee et al., 2025). Using QIIME 2 and R, statistical analysis was conducted to compare microbial communities across experimental groups.

## Results

### Isolation and identification of biomarker strains

*Bifidobacterium* has been identified as a biomarker in healthy individuals compared to *Prevotella* in gout patients through a metagenomic approach (ul-Haq et al., 2022). In this study, our search was to isolate *Bifidobacterium* from healthy stool samples and to evaluate their potential therapeutic effect in mice models. A total of 17 samples were collected for analysis, all showing bacterial growth. From these samples, we selected a total of 59 bacterial colonies based on observed color changes and morphology (Table S1). To further confirm and validate their taxonomy, selected single colonies were sent for 16S rRNA gene sequencing, in which 17 isolates were identified as *Bifidobacterium* strains (Table S2), in which the most abundant strains were *B. longum* and *B. bifidum*. Initially, the strains were identified through 16S rRNA gene sequencing. The *B. longum* strain used for treatment in mice exhibited a potential therapeutic effect against gout and was further identified by whole-genome sequencing as *B. longum* PMC72.

### Whole-genome analysis result of the strain

**Fig. 1.**
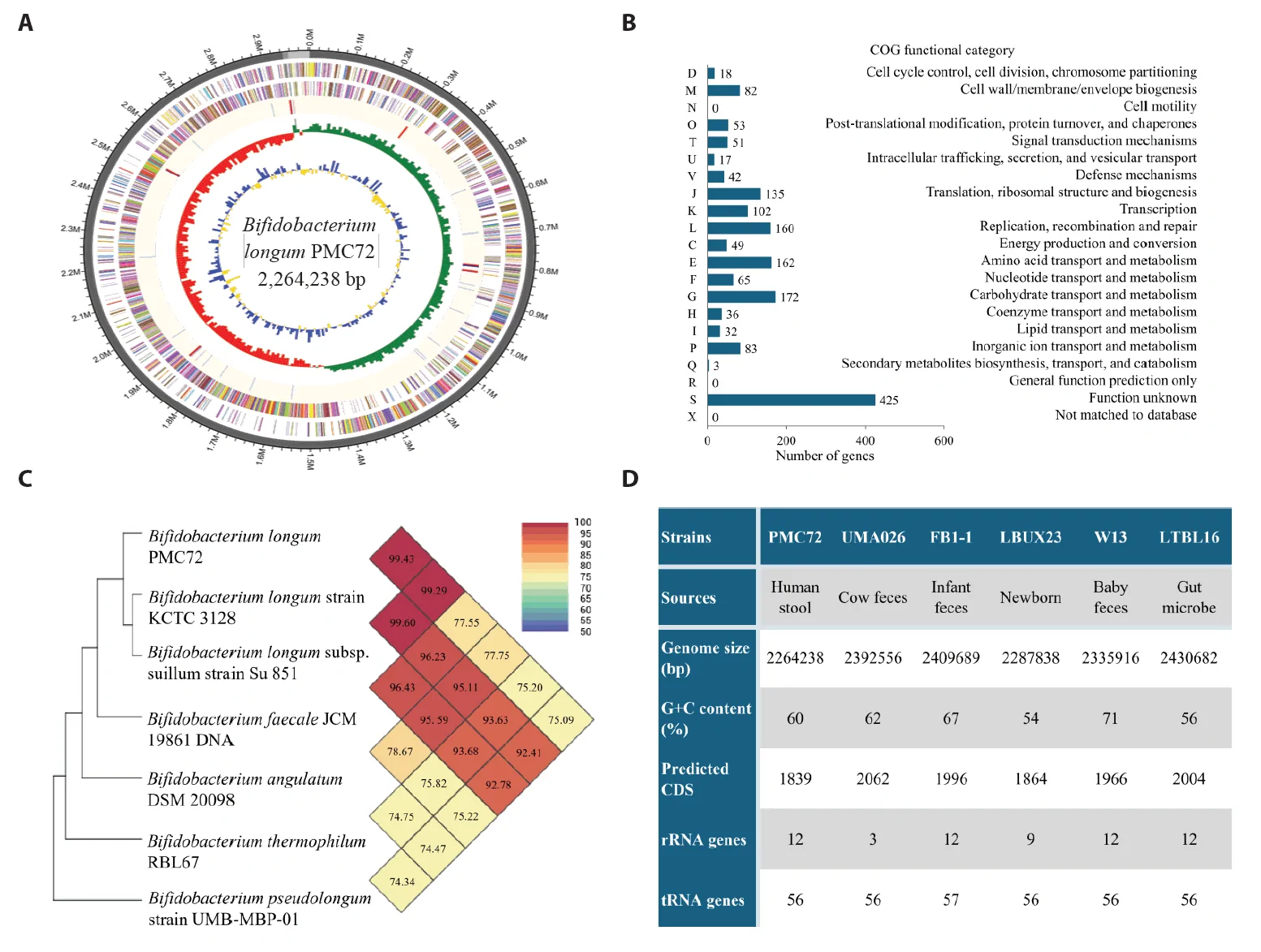
WGS analysis and comparative genomics of *B. longum* PMC72. This figure offers a comprehensive genomic overview of PMC72, underscoring its structural and functional genomic characteristics. (A) Displays a circular genome map of *B. longum* PMC72, with color-coded gene clusters categorized by COG. tRNA and rRNA genes are marked in red and blue, respectively, while GC skew (yellow/blue) and GC content (red/green) are indicated in the inner circle. (B) The functional classification of the genes reveals the relative abundance of proteins assigned to COG (Clusters of Orthologous Groups) families, with biological functions identified for 1687 proteins, while the roles of 425 CDSs remain unknown. (C) An OrthoANI comparison demonstrates a 99.43% similarity between *B. longum* PMC72 and *B. longum* KCTC 3128, confirming their species-level identity. (D) Comparative genomics between PMC72 and related *B. longum* strains (KCTC 3128, K15, NBRC 114370, and JCM 19995) utilizes the table to illustrate genome size, G + C content, CDS count, and quantity of rRNA and tRNA genes, showcasing the functional genomic differences that make PMC72 a unique strain.

The results of the whole-genome sequencing analysis for the strain are depicted in Fig. 1. The genome is composed of a single circular chromosome spanning 2,264,238 bp, housing 1,839 coding DNA sequences (CDSs) (Fig. 1A). Among these, 1,687 predicted CDSs were functionally classified based on the Clusters of Orthologous Groups (COGs) (Fig. 1B). Biological functions were assigned to 1,401 of these predicted proteins, while 528 CDSs exhibited homology to conserved proteins with unidentified functions in other organisms. Additionally, 25 hypothetical proteins did not exhibit matches with any known proteins in the database. Utilizing the OrthoANI method, a similarity analysis was conducted for strains exhibiting high similarities in 16S rRNA analysis, employing the entire genome sequence data of the strain depicted in Fig. 1C. The analysis affirmed a 99.43% similarity between the strain and *B. longum* strain KCTC 3128 and *B. longum* subsp. Su 851, highlighting their subspecies-level distinction (Lawley et al., 2017). Furthermore, its similarities with other species within the *Bifidobacterium* genus, namely *B. faecale*, *B. angulatum*, and *B. thermophilum*, were 77.55%, 77.55%, and 75.20%, respectively. Furthermore, a comparative analysis of genome size, G + C content, CDS, rRNA, and tRNA gene counts between the probiotic strain PMC72 and related *B. longum* strains is presented in Fig. 1D. These findings provide comprehensive genomic insights into the strain, confirming its high genetic similarity to *B. longum* and distinguishing its genomic features from other species within the *Bifidobacterium* genus.

### Evaluation of the therapeutic effects of PMC72 on gout-related symptoms and renal pathology

**Fig. 2.**
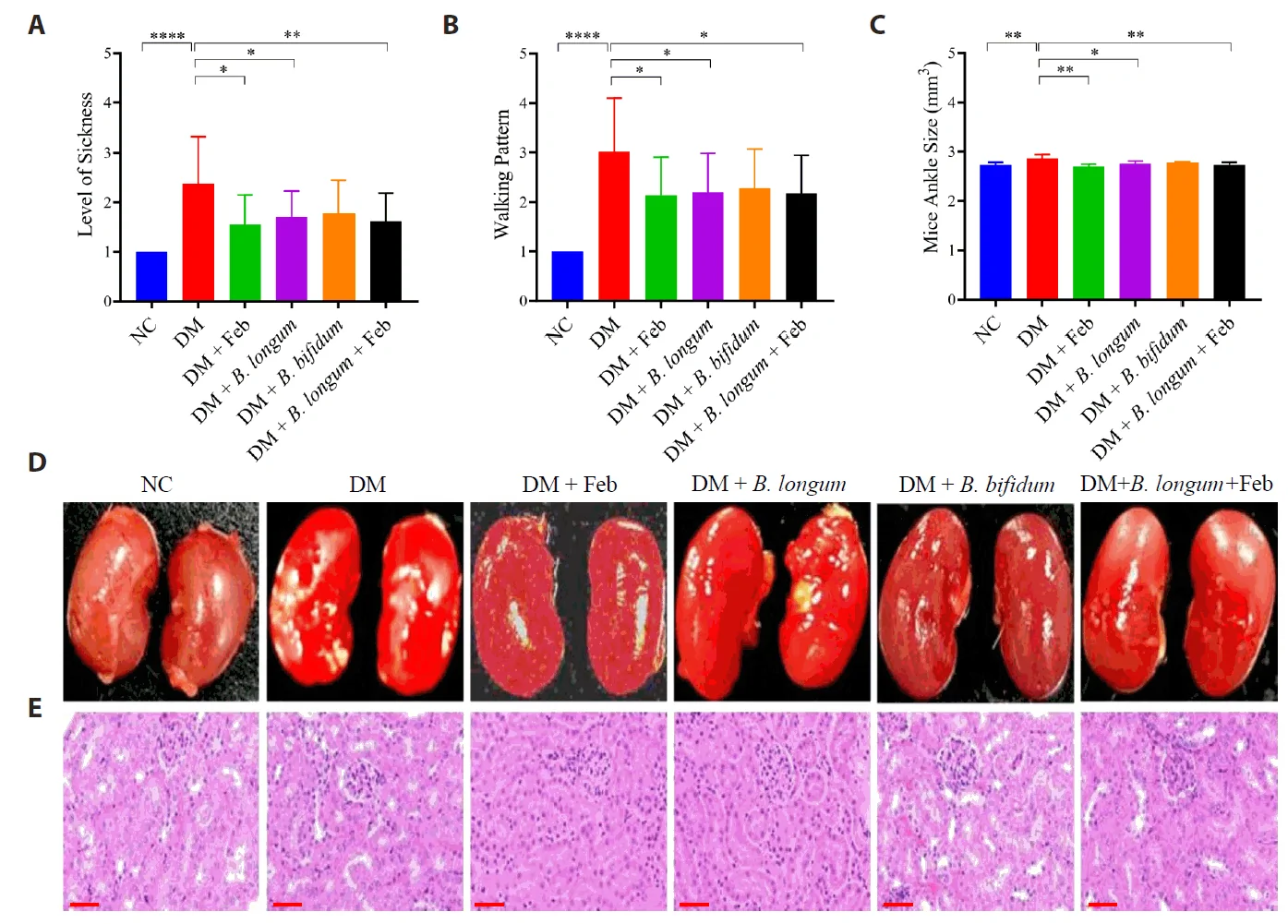
This figure demonstrates the impact of microbiome-based treatments on both behavioural and physiological markers in a gout mice model. Following PO-induction, mice display significantly increased sickness behaviour scores, altered walking patterns, and enlarged ankle size. Treatment with Febuxostat and *B. longum* PMC72 significantly improved (A) sickness level, (B) walking pattern, and (C) ankle inflammation after 10 days when compared to the DM group. Co-treatment with PMC72 and Febuxostat further enhanced these improvements. (D) Kidney images taken immediately after dissection showed a healthier kidney appearance in the probiotic-treated groups compared to the DM group. (E) Histopathological examination of kidney tissues stained with H&E (Scale bar = 50 µm) revealed that the NC group maintained normal glomerular and tubular structures. In contrast, the DM group exhibited glomerular tuft shrinkage and increased cellular infiltration in the periglomerular and interstitial regions. Treatment with Febuxostat and PMC72, both individually and in combination, maintained glomerular integrity and enhanced lumen density relative to the DM group. Statistical significance was analyzed using GraphPad Prism 9.1.1 using one-way ANOVA.

The therapeutic effects of PMC72 on gout-related symptoms and renal pathology in a mouse model were evaluated through sickness assessment, walking pattern and ankle size measurements, and renal histopathological analysis (Fig. 2).

The analyzed data showed that the sickness behaviour score has significantly (*p* < 0.0001) increased in mice treated with potassium oxonate compared to the NC (Fig. 2A). Subsequently, when the diseased mice were treated with Feb (*p* < 0.05) or *B. longum* (*p* < 0.05), the level of sickness behaviour score decreased significantly, whereas treated with *B. bifidum* had no significant impact on mice health. Furthermore, when the diseased mice were treated with Feb and *B. longum* together, the sickness behaviour score was significantly reduced (*p* < 0.01). The walking pattern of mice was worsened after the PO injection, a visible swelling was noticed, and the dysfunction index was calculated at 3.02 (Fig. 2B). In the febuxostat-treated groups, the dysfunction index was 2.14 (*p* < 0.05), while *B. longum* treated group was 2.19 (*p* < 0.05). In both cases, we see a significant reduction in dysfunction. Combined treatment of the drug and PMC72 also significantly (*p* < 0.05) reduced the level of dysfunction. Ankle size measurement of each mouse was conducted using vernier calipers at various stages, including before treatment, during PO induction, and after treatment. The average ankle size in the control group was 2.73 mm^3^, whereas in the DM, it significantly increased to 2.86 mm^3^ (*p* < 0.05) (Fig. 2C). The Febuxostat and *B. longum* treated group showed sizes of 2.70 mm^3^ (*p* < 0.01) and 2.76 mm^3^ (*p* < 0.05), respectively meaning a significant reduction of swelling. In the *B. bifidum*-treated group, the reduction was not significant again when combined treatment with *B. longum* healed significantly (*p* < 0.01).

Histopathological analysis of mice kidney tissue was conducted to observe the density of glomerular tissue. Initially, images of the kidney were taken immediately after dissection to observe changes in different groups upon treatment (Fig. 2D). Examination of kidney structures in the NC, as of Fig. 2E, revealed normal glomerular and tubular epithelial features, with neatly arranged brush borders in renal tubules. In contrast, mice treated with PO exhibited evident tubular lesions characterized by indistinct boundaries between adjacent proximal tubule cells, tubular expansion, and aggregation of inflammatory cells around renal blood vessels. Post-treatment with *B. longum* and/or Febuxostat demonstrated varying attenuation in renal tubular injury and inflammatory cell infiltration.

### Biochemical and oxidative stress analysis of *B. longum* PMC72 in hyperuricemic mice

**Fig. 3.**
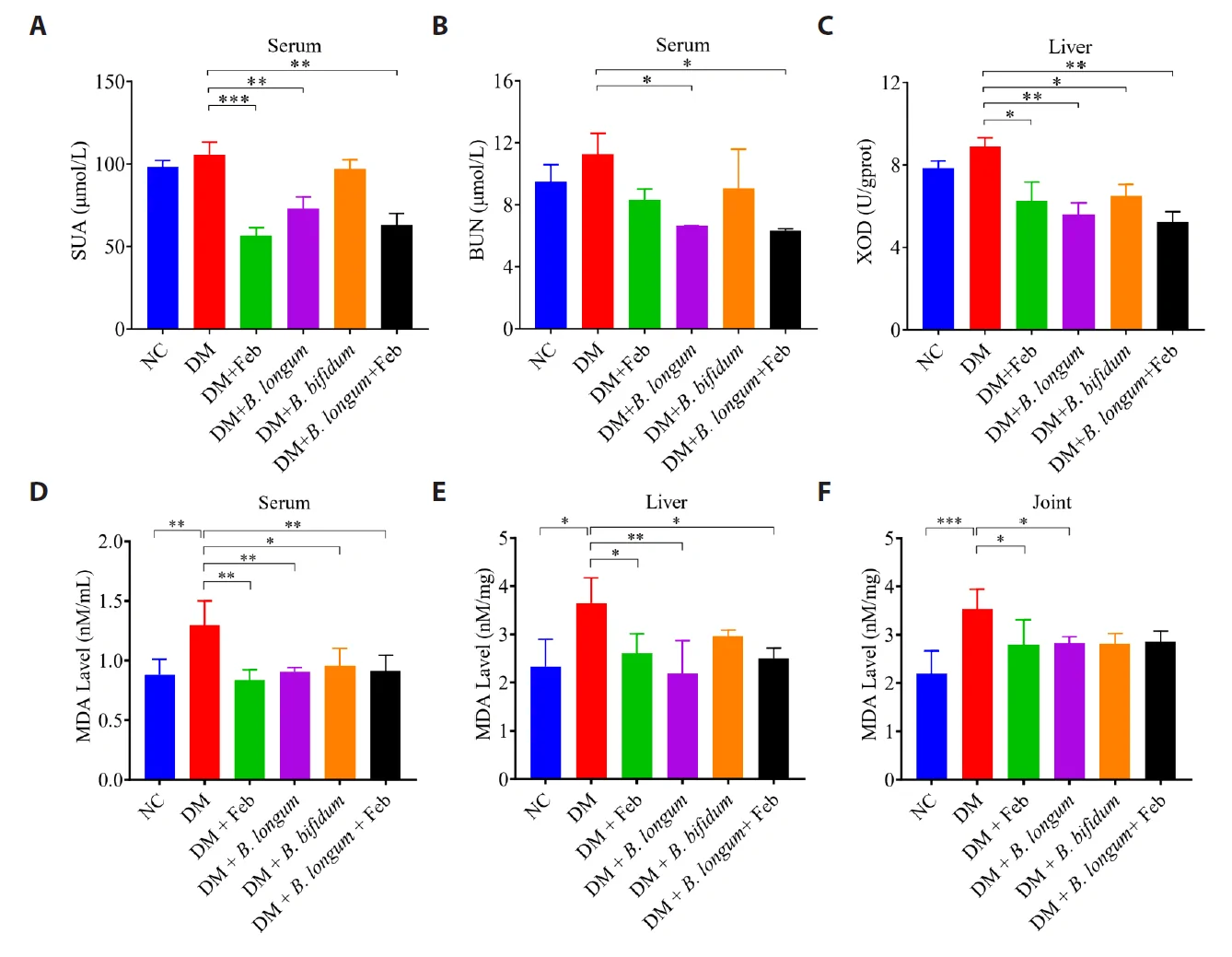
Activity of *B. longum* PMC72 in biochemical and oxidative parameters in hyperuricemic mice. This figure illustrates the effects of PMC72 on key biochemical and oxidative stress markers in hyperuricemic mice, either alone or in combination with Febuxostat. Serum and liver samples were tested for (A) Serum Uric Acid, (B) Blood Urea Nitrogen, and (C) Xanthine Oxidase, with findings indicating a significant decrease in all parameters following PMC72 treatment, suggesting its potential therapeutic impact in mitigating hyperuricemia. Oxidative status was evaluated by measuring malondialdehyde (MDA) levels in (D) serum, (E) liver, and (F) joint samples, revealing a significant increase following PO induction. However, PMC72 treatment significantly reduced MDA levels across all tissues. Combined treatment with PMC72 and Febuxostat yielded similar improvements across all parameters. Values are presented as mean ± standard deviations, with statistical significance determined using GraphPad Prism 9.1.1 using one-way ANOVA.

To assess *B. longum*’s ability to inhibit uric acid (UA) production in mice, various parameters including SUA, BUN, and XOD activities were measured. As depicted in Fig. 3A, PO administration led to an increase in SUA concentration compared to the NC group, leading to the construction of the hyperuricemia model in DM. Oral administration of Febuxostat for 10 days decreased SUA (*p* < 0.001) in mice. Following *B. longum* treatment, SUA levels also decreased significantly (*p* < 0.01). At the same time, *B. bifidum* showed no significance in reducing SUA levels, which remains almost identical to the PO-administered group. Combined treatment also resulted in significant SUA reduction (*p* < 0.01), indicating synergistic efficacy between *B. longum* and drugs.

Serum levels of BUN, a crucial indicator of renal function, were measured in Fig. 3B. As anticipated, BUN values in the DM increased compared to the NC group, indicating kidney impairment. However, both Febuxostat and *B. longum* effectively reversed the PO-induced increase in BUN levels. While in the DM group, blood urea nitrogen level was higher, Febuxostat, *B. longum* (*p* < 0.05), and *B. bifidum* treatment decreased the level. Again, combined treatment furthermore reduced BUN level (*p* < 0.05).

Figure 3C demonstrates that after injection with PO, the hepatic XOD level of the DM group was higher compared to normal mice. However, supplementation with Febuxostat (*p* < 0.05), *B. longum* (*p* < 0.01), *B. bifidum* (*p* < 0.05), and combined treatment (*p* < 0.01) significantly reduced elevated XOD activities in the liver.

The levels of malondialdehyde in the serum, liver, and ankle joint were explored. There was a significant increase in malondialdehyde level after induced with PO. While a significant reduction in MDA levels in the serum samples when treated with either Feb (*p* < 0.01) or *B. longum* (*p* < 0.01) compared to the DM group (Fig. 3D). Moreover, the combined treatment of Feb and candidate strain resulted in a significant reduction in MDA levels compared to the diseased group. A significant reduction in MDA was also noticed when diseased mice were treated with probiotic *B. bifidum* (*p* < 0.05). However, this reduction was not as high as in *B. longum* or Feb. In the case of the liver, treating diseased mice with Feb or *B. longum* significantly lowered MDA levels (*p* < 0.05 and *p* < 0.01, respectively) compared to the DM group (Fig. 3E). Combining Feb and candidate strain treatments also significantly reduced MDA levels (*p* < 0.05). Moreover, the *B. bifidum* treatment lowered MDA levels, though not as effectively as the *B. longum* or Feb treatment. Additionally, the data showed a significant reduction in MDA levels in the ankle joint samples treated with either Feb (*p* < 0.05) or *B. longum* (*p* < 0.05) compared to the DM group (Fig. 3F). Furthermore, the combined treatment of Feb and *B. longum* strain resulted in a notable decrease in MDA levels compared to the DM group, although it could not reach a significant level. Treatment with *B. bifidum* did not show a significant reduction compared to the DM group.

### Inflammatory gene expression analysis

**Fig. 4.**
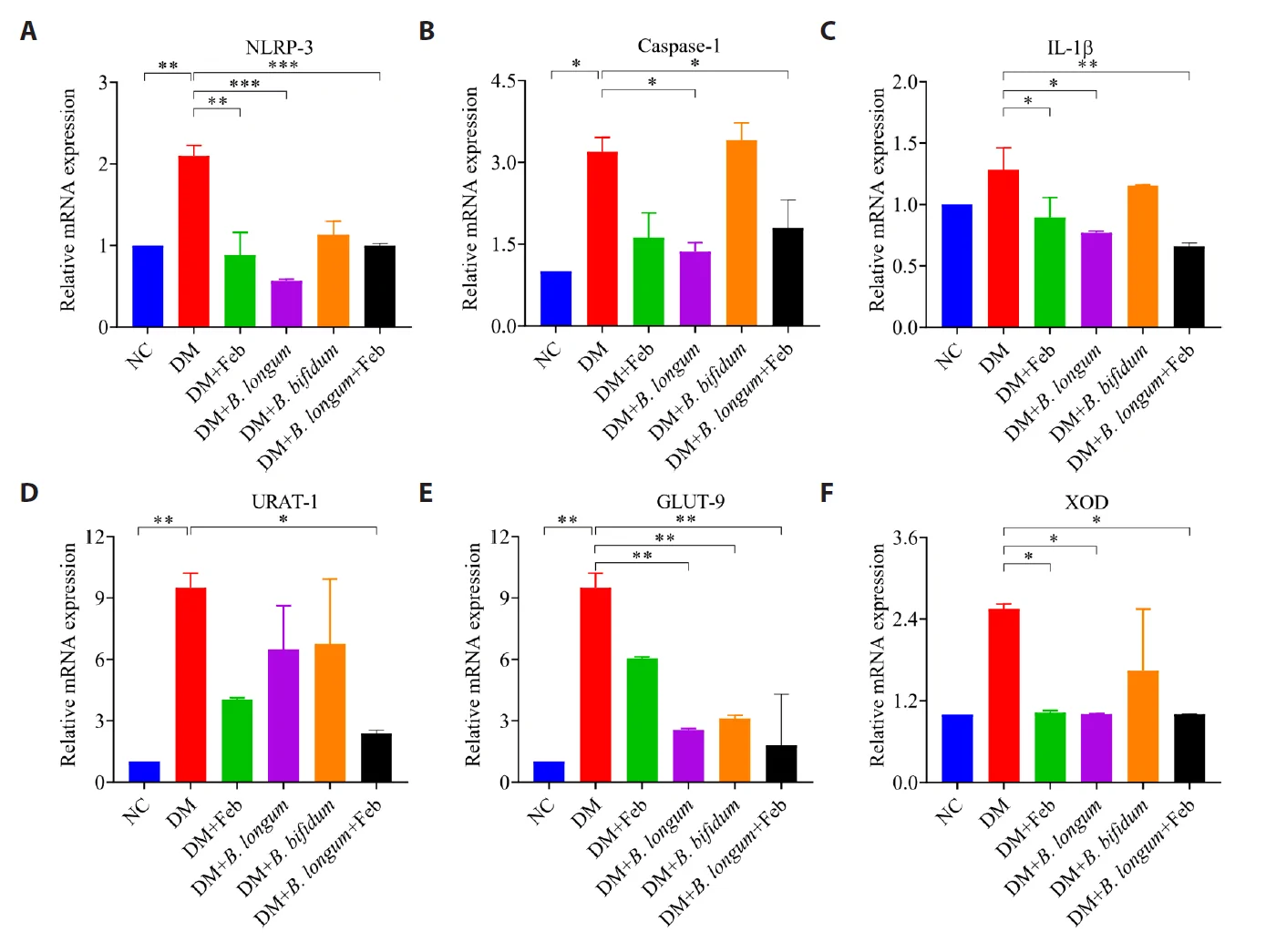
Effect of therapeutic interventions on gene expression related to inflammation and UA transportation in a hypouricemic mice model. This figure displays the relative expression levels of genes associated with inflammation and uric acid transport in kidney samples from six treatment groups. Panels (A–C) illustrate inflammatory gene expression, (D–E) detail renal UA transporter genes, and (F) shows xanthine oxidase synthetase gene expression. In all cases, DM exhibits elevated gene expression. At the same time, microbiome therapeutic PMC72 treatment reduced the levels of inflammation, transporters, and enzymes, indicating that the probiotic can effectively reduce uric acid catabolism, resulting in lower uric acid levels in the kidney. Values are expressed as mean values and standard deviations. Statistical significance was analyzed using Graph Pad Prism 9.1.1 using one-way ANOVA. The primers used are presented in Table S3.

Gene expressions regarding inflammation and UA transportation were analyzed after the treatment of gouty mice. Activation of the NLRP3 inflammasome was observed in the kidneys of mice with gout arthritis. As depicted in Fig. 4A–4C, there was a significant increase in the expression of inflammatory genes in the PO-induced mice model (DM) compared to NC (*p* < 0.05). Treatment with Febuxostat and *B. longum*, both individually and in combination, significantly reduced the production of NLRP3, caspase-1, and IL-1ß compared to DM. Treatment with Febuxostat reduced the production of NLRP3 (*p* < 0.01), caspase-1 (*p* < 0.05), and IL-1ß (*p* < 0.05) while PMC72 reduced the production of NLRP3 (*p* < 0.001), caspase-1 and IL-1ß (*p* < 0.05). No significant reduction in gene expression was observed while comparing the *B. bifidum*-treated group with DM. The combination of Drug and PMC72 again significantly reduced the expression of the inflammatory genes NLRP3, caspase-1, and IL-1ß.

Figure 4D and 4E shows the impact of treatment using both drug and microbiome therapeutics on the mRNA expression of GLUT9 and URAT1 in mice induced with PO. PO challenge markedly increased the expression levels of kidney URAT1 and GLUT9 genes compared to the NC group. However, treatment with Febuxostat, notably decreased the elevated mRNA levels of URAT1 and GLUT9 in mice with gout. However, in the case of probiotic *B. longum* and *B. bifidum*, there is no significant change in URAT-1 expression, but in GLUT-9, the reduction (*p* < 0.01) is detected.

We have previously confirmed the reduction of XOD for liver samples using an Elabscience Kit. Here, we checked the reduction of XOD gene expression of mice mRNA. The results in Fig. 4F demonstrated that in the DM group, the expression levels of XOD genes were upregulated by PO compared to NC. Both drug treatment and *B. longum*, alone or in combination, showed a reduction (*p* < 0.05) in gene expression, indicating reduced UA production after treatment. The changes after *B. bifidum* treatment have yet to be significant again.

### NGS-based gut dysbiosis analysis

**Fig. 5.**
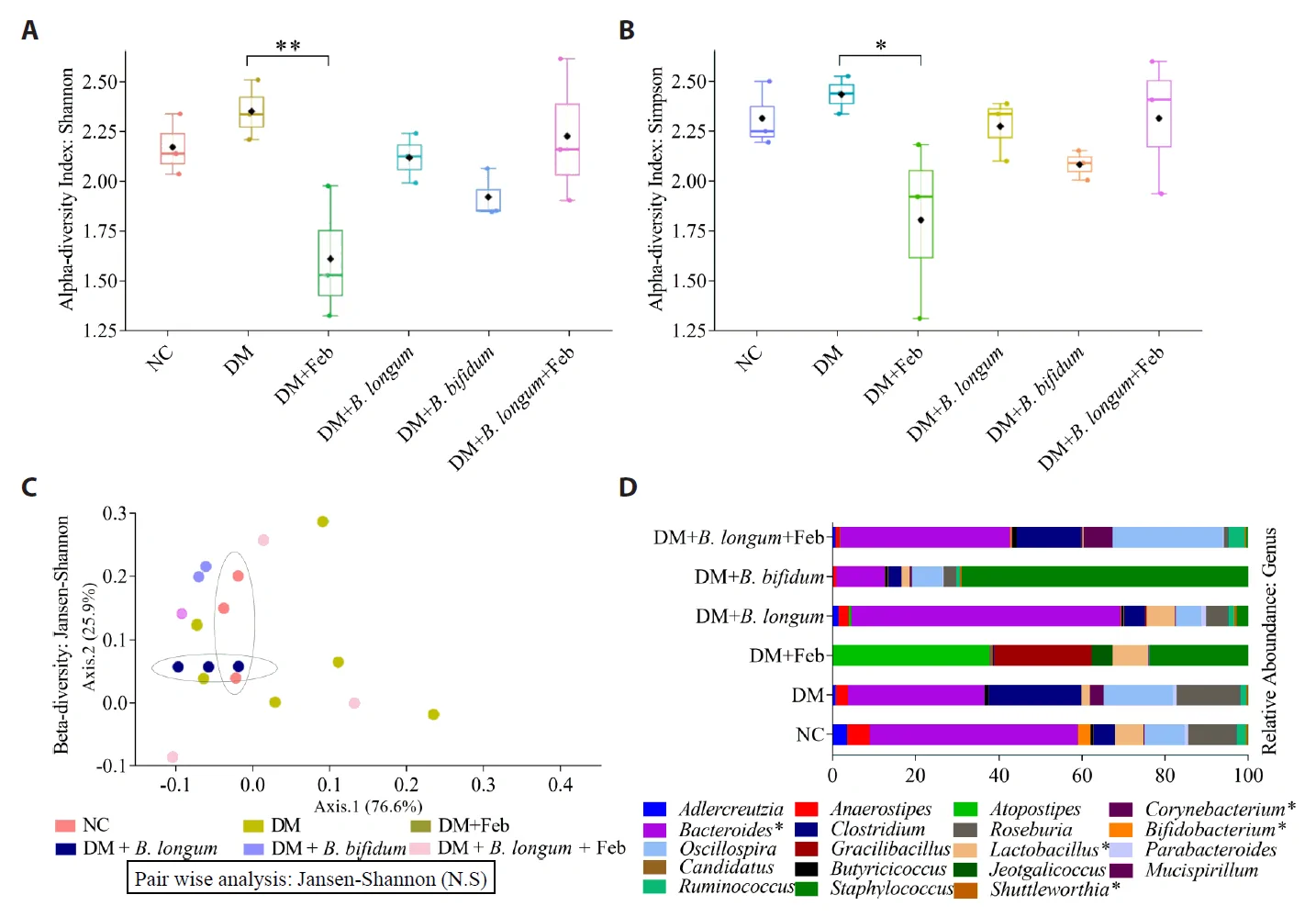
NGS-based gut dysbiosis analysis in a gout disease model. To evaluate the impact of *B. longum* PMC72 on gut microbiota dysbiosis in a gout-induced mouse model, we conducted high-throughput sequencing analysis to assess alpha and beta diversity and microbial composition across different treatment groups. (A–B) Alpha diversity analysis (Shannon and Simpson Index) shows significant differences between groups (*p* < 0.05) using the Kruskal-Wallis test. Pairwise comparisons revealed that while PO and febuxostat treatment change the microbial composition, treatment with *B. longum* and combined treatment do not. (C) Beta diversity analysis using Jensen-Shannon divergence through PCoA reveals distinct clustering patterns, with the NC and DM + *B. longum* groups exhibiting similar microbial community structures. (D) Relative abundance analysis at the genus level highlights microbial composition across the experimental groups. Notably, the DM + *B. longum* has higher percentage of *Lactobacillus* (6.6%), *Bacteroides* (64.1%), and *Oscillospira* (6.2%) closely resembling the NC group.

NGS-based analysis of the gut microbiome in the gout disease model revealed significant differences in microbial diversity and composition among the experimental groups (Fig. 5). Alpha diversity analysis (Fig. 5A and 5B) revealed significant changes in microbial composition in the DM and Feb-treated groups (*p* < 0.05), with similar alterations observed in the NC group. However, PMC72 treatment, either alone or combined with febuxostat, preserved diversity, closely resembling the NC group. Beta diversity analysis using Jensen-Shannon divergence visualized through Principal Coordinates Analysis (Fig. 5C). The plot illustrates the clustering of microbial communities based on compositional differences across groups. The NC and DM + *B. longum* groups exhibit closer clustering, indicating a microbial composition that is more similar to other groups. Relative abundance analysis at the genus level (Fig. 5D) highlighted substantial dysbiosis in the DM group, with reduced beneficial genera such as *Lactobacillus* (2.1%) and *Bacteroides* (32.3%) and increased opportunistic microbes (Louisiana Office of Public Health, 2015) like *Clostridium* (22%). Febuxostat treatment partially restored beneficial genera but was associated with an unusual rise in *Staphylococcus* (23%, compared to < 1% in NC), contributing to dysbiosis. In contrast, PMC72 treatment restored microbial composition to near-homeostasis, with beneficial genera like *Lactobacillus* (6.6%), *Bacteroides* (64.1%), and *Oscillospira* (6.2%) closely resembling the NC group. These findings suggest that PMC72 effectively modulates the gut microbiota, preventing dysbiosis in the gout model.

## Discussion

Hyperuricemia, a disorder stemming from abnormalities in purine metabolism, presents a significant global health challenge (Kuo et al., 2015). The prevalence of hyperuricemia is influenced by factors such as lifestyle, diet, genetics, stress, and overall health (Dalbeth et al., 2019). However, traditional treatments for hyperuricemia have notable drawbacks, including allopurinol hypersensitivity syndrome, increased cardiovascular risk with Febuxostat, and hepatotoxicity and kidney stone formation with benzbromarone, underscoring the need for safer alternatives. While dietary modifications (e.g., limiting purine intake and reducing alcohol consumption) can help manage hyperuricemia, they are generally insufficient to achieve therapeutic reductions in serum uric acid levels (Dalbeth et al., 2021). In light of these challenges, a growing focus is on modulating uric acid metabolism through gentle microbial therapeutic interventions. Research suggests that probiotic supplements hold promise in effectively mitigating uric acid accumulation. For instance, studies by Wu et al. (2021) demonstrated the urate-lowering effects of *Limosilactobacillus fermentum* JL-3 in mice, alongside its modulation of gut microbial composition. Similarly, investigations conducted by Kuo et al. (2021) showcased the ability of *Lactobacillus reuteri* TSR332 and *Lactobacillus fermentum* TSF331 to stabilize SUA levels and prevent hyperuricemia in mice models. Recent advancements have further expanded the scope of probiotic interventions, with live and heat-treated *Akkermansia muciniphila* emerging as a potential modulator of uric acid metabolism in mice (Zhang et al., 2022) Additionally, prophylactic administration of *Lactobacillus brevis* DM9218-A has shown promise in reducing SUA concentrations in hyperuricemia-affected mice (Li et al., 2014). Thus, probiotics with UA-lowering ability might be a promising strategy for treating hyperuricemia.

Numerous reports have been published as the potential of microbiome therapeutics in gout continues to emerge (Seo et al., 2024). However, we developed a therapeutic approach based on clinical metagenomic biomarkers through reverse translational research instead of traditional screening methods. This process begins with NGS analysis of samples from healthy individuals and patients with specific diseases to identify biomarker strains. The identified strains were isolated from clinical samples, followed by efficacy and safety tests conducted to assess their therapeutic potential. This process concludes with a standard drug development pipeline involving GLP (Good Laboratory Practice) toxicology tests and GMP (Good Manufacturing Practice)-based clinical trials and regulatory approval, ensuring safety and efficacy. This framework represents a systematic approach that surpasses traditional screening methods for developing microbiome-based therapeutics. Our prior study performed 16S rRNA gene-based microbiota analysis on 65 stool samples, including 17 from healthy controls and 48 from febuxostat-treated gout patients (ul-Haq et al., 2022). The results showed reduced alpha diversity in bacterial communities across the healthy control, controlled, and uncontrolled groups, while beta diversity analysis revealed significant differences in bacterial community structures. The biomarkers were discovered through a combination of Taxonomic data, LEfSe analysis and qPCR assays. LEfSe analysis was used to identify taxa with significant differences in effect sizes between healthy controls and the two gout groups, with genera such as *g-Bifidobacterium* and *g-Prevotella* emerging as key biomarkers. Specifically, *g-Bifidobacterium* was more prominent in healthy controls, while *g-Prevotella* was most abundant in the uncontrolled gout group. These findings were validated using qPCR assays, which confirmed the distinct population levels of these genera in each group. The data suggest that *g-Bifidobacterium* may serve as a potential biomarker for healthy group, while *g-Prevotella* for gout patients. Based on these findings, *Bifidobacterium* was deemed a promising candidate as a microbiome therapeutic targeting gout. We subsequently isolated a wide range of *Bifidobacterium* species from the analyzed healthy stool samples. Among the isolates, *B. longum* and *B. bifidum* were the most predominant and were further studied in vivo to evaluate their efficacy. *B. longum* demonstrated significant in vivo effectiveness and was identified as *B. longum* PMC72 through WGS.

For gout inducing in mice PO was administered intraperitoneally. This method is employed to initiate hyperuricemia in mice, mimicking certain aspects of human gout, allowing researchers to study the pathophysiology and potential therapeutic interventions for this inflammatory condition (Tang et al., 2017). Potassium oxonate also acts as a specific competitive uricase inhibitor (Tang et al., 2017), significantly increasing UA concentration. As gout is a disease of visible discomfort, it is important to observe the movement of mice. Before sacrificing the mice, we assessed their sickness behavior score, walking pattern, and ankle size, which showed a significant increase in DM compared to NC, confirming the successful establishment of gout in the mice. After the treatment of probiotic *B. longum*, all the parameters have been significantly improved in the treated group compared to the diseased ones. These findings align with the previous findings by El-Dein et al. (2023).

Histopathological analysis examines tissue changes at the microscopic level, providing crucial insights into inflammation and structural alterations associated with diseases like gout (Yan et al., 2023). The link between impaired renal function and elevated SUA levels in patients with asymptomatic hyperuricemia or gout has been well-documented. Several mechanisms have been proposed to explain the relationship between hyperuricemia and chronic kidney disease, including the effects of urate crystals, activation of the renin-angiotensin-aldosterone system, inhibition of nitric oxide synthesis, and stimulation of intracellular oxidative stress (Lee et al., 2021). In this study, it is essential to assess how probiotic treatment impacts kidney health in gout-induced mice by identifying tissue improvements and reductions in inflammation. The treated group showed downregulation of inflammation and dysfunction. This pattern was also observed in histopathological hematoxylin and eosin (H&E) micrographs, indicating that PMC72 could improve kidney inflammation in mice. Approximately 70% of uric acid is excreted by the kidneys. Consequently, elevated serum uric acid levels can induce structural changes in renal components such as the glomerulus, Bowman’s capsule, and tubules (Ohno, 2011). Based on the findings, it is evident that the administration of PMC72 effectively reduced the dilation of proximal tubule cells and normalized Bowman’s capsule space, highlighting its potential to improve renal health in hyperuricemic conditions.

Xanthine oxidoreductase (XOR) is a crucial enzyme found predominantly in the liver, essential for catalyzing the final steps of purine breakdown. XOR exists in two forms: xanthine dehydrogenase (XDH) and xanthine oxidase (XOD) (Vorbach et al., 2003), with XOD being particularly involved in uric acid production. Elevated XOD levels can result in excessive UA production, making XOD inhibition a key strategy for gout treatment. Allopurinol and febuxostat are common drugs used as classic XOD inhibitors, but they sometimes have undesirable side effects. Our findings, consistent with previous reports (Cao et al., 2022; Vieira et al., 2015), indicate that *B. longum* significantly reduces SUA levels and liver XOD activities. Moreover, inadequate renal excretion of uric acid (UA) contributes to hyperuricemia (Zhang et al., 2018). The kidney, a key regulator of SUA levels, plays a crucial role in maintaining urate balance by managing its secretion and reabsorption in the renal tubules. In our study, PO supplementation notably increased BUN and SUA levels compared to the NC group, indicating renal dysfunction. In contrast, treatment with *B. longum* and febuxostat significantly reduced elevated SUA and BUN levels compared to the DM group.

The pathogenesis of gout is multifactorial and not fully understood, but oxidative stress and the resulting damage to proteins and lipids are commonly observed (Acharya et al., 2015). Oxidative stress occurs when there is an imbalance in the prooxidant-antioxidant balance, favoring prooxidants, which triggers various pathophysiological processes (Rahal et al., 2014). Lipid peroxidation is a self-propagating process in which fatty acids in cell membranes undergo oxidation, producing a range of byproducts, including malondialdehyde. As such, MDA levels are frequently used to assess lipid peroxidation. This study investigated the impact of *Bifidobacterium* strains on MDA levels in serum, liver, and ankle joints, demonstrating notable reductions in MDA levels. MDA is a reactive aldehyde and a byproduct of lipid peroxidation, serving as a biomarker for oxidative stress. In gout, a form of inflammatory arthritis caused by elevated uric acid levels leading to urate crystal formation in joints, oxidative stress significantly contributes to inflammation and tissue damage. Elevated MDA levels in gout indicate increased oxidative stress associated with this inflammation. Our findings demonstrate that the gout model exhibited elevated MDA levels compared to the control group, suggesting increased oxidative stress in these tissues. This aligns with previous research highlighting the role of oxidative stress in gout pathogenesis (Palygin et al., 2021). However, MDA levels decreased following candidate probiotic administration, which aligns with previous findings showing the antioxidant properties of specific probiotic strains that can influence systemic inflammation (Rezazadeh et al., 2021; Rochat et al., 2007).

Numerous studies have indicated that inflammation is a common pathological characteristic of hyperuricemia and plays a crucial role in its progression (Zhang et al., 2018). Gut inflammation plays a significant role in the development of gout arthritis (Dalbeth et al., 2019). Pro-inflammatory cytokine release is central to kidney inflammation, with the NLRP3 inflammatory pathway being a key regulator of inflammation (Wu et al., 2017). The NLRP3 inflammasome, comprising the NLRP3 receptor and caspase-1 effector, is an intracellular complex implicated in inflammation regulation. Growing evidence suggests that elevated urate levels directly activate the NLRP3 inflammasome, leading to the maturation and secretion of IL-1β, which contributes to kidney inflammation and dysfunction (Zhang et al., 2018). Our findings demonstrated activation of the NLRP3 inflammasome in hyperuricemic mice, as indicated by increased renal mRNA expression of NLRP3, caspase-1, and IL-1β. Remarkably, Febuxostat, *B. longum*, and combined treatment significantly suppressed these increases. The kidney is the primary organ responsible for excreting uric acid, with approximately two-thirds of UA excretion occurring through the kidneys. Renal UA reabsorption transporters such as GLUT9 and the excretory transporter URAT1 play crucial roles in this process (Hoque et al., 2020; Maiuolo et al., 2016). In our study, we observed increased expressions of GLUT9 and URAT1 in the DM group. However, oral administration of *B. longum* reduced GLUT9 expression to normal levels, while combined therapy significantly reduced both.

In the study of gut health and disease, metagenomic analysis of dysbiosis is essential for evaluating the effects of probiotics (Castilho et al., 2023), such as PMC72, on microbiome composition. Dysbiosis, an imbalance in microbial communities, has been linked to a variety of inflammatory and metabolic disorders, including gout (Shirvani-Rad et al., 2023; Xi et al., 2019). In gout, dysbiosis may exacerbate disease symptoms and inflammation, making it important to assess the microbiome's response to probiotic treatments that could rebalance microbial diversity. Understanding these microbial dynamics is crucial for advancing gout therapies, as probiotics may prevent or alleviate dysbiosis and reduce disease severity. Our study provides valuable insights into the effects of febuxostat and *Bifidobacterium* strains, particularly PMC72, on the gut microbiome in a gout disease model. Sample sizes and species richness in 16S rRNA sequencing were analysed (Fig. S2), revealing gout-induced gut dysbiosis with reduced Shannon diversity and a shift in microbial composition, with the DM group showing a decline in beneficial bacteria such as *Lactobacillus* and *Bacteroides* and an increase in opportunistic genera like *Clostridium*. Although Febuxostat partially restored beneficial genera, it caused an unexpected rise in *Staphylococcus*, a genus typically found at negligible levels in healthy controls, underscoring its limited efficacy in addressing dysbiosis. In contrast, the PMC72 treatment effectively preserved microbial diversity and restored the gut microbiome composition to near-homeostasis, with the DM + *B. longum* group displaying relative abundances of beneficial genera such as *Lactobacillus*, *Bacteroides*, and *Oscillospira*, closely resembling the NC group and significantly reducing pathogenic genera. These findings suggest that PMC72 has the potential to stabilize the gut microbiota and prevent the dysbiosis associated with gout pathology. By preserving microbial diversity and shifting the microbiome composition toward a healthier state, PMC72 may mitigate the inflammatory and metabolic disruptions linked to gout. Future studies should explore the long-term effects of PMC72 on clinical outcomes in gout patients and investigate the mechanisms underlying its microbiome-modulating effects, paving the way for probiotic-based strategies as adjunct therapies in gout management.

While existing gout medications often have limitations, including side effects and limited efficacy, our study takes a novel reverse translational approach to address these challenges. Building on prior research linking gut microbiota to gout and identifying *Bifidobacterium* as a key biomarker, we investigated multiple strains of *Bifidobacterium* in a gout mouse model. PMC72 demonstrated promising therapeutic effects, including reduced inflammation, improved kidney function, and lower uric acid levels. These findings position PMC72 as a strong candidate for microbiome-based therapeutic development for gout, offering a safer and more targeted alternative to conventional treatments. However, several challenges remain before PMC72 can be translated into clinical applications. Regulatory approval will require extensive safety and efficacy evaluations, including FDA review and GRAS or QPS designation, followed by rigorous clinical trials (Misra and Raghuwanshi, 2022). Determining an optimal and standardized human dosage remains a key hurdle (Ahmed et al., 2024), as differences in gut microbiota composition, metabolism, and immune responses across populations may influence treatment outcomes. Future research will focus on conducting well-controlled clinical trials to establish the appropriate administration route, dosage, and duration of PMC72 therapy, ultimately advancing it toward clinical use for gout treatment.

## Conclusion

In conclusion, a reverse translational therapeutic study of PMC72 demonstrated significant efficacy for gout management in a potassium oxonate-induced mouse model. PMC72 alleviated gout symptoms, including joint inflammation and renal dysfunction, by reducing serum uric acid, oxidative stress, and hepatic xanthine oxidase activity while downregulating renal uric acid transporters to enhance urate excretion. Furthermore, PMC72 restored gut microbiome balance disrupted by hyperuricemia, offering a distinct advantage over conventional treatment with Febuxostat, mainly when combined. These findings position PMC72 as a promising and holistic microbial therapeutic for gout, addressing the disease’s systemic and microbiome-related aspects. Finally, this study outlines a stepwise methodological approach for developing and evaluating the biomarker strain *B. longum* PMC72 as a gout therapeutic, presenting a comprehensive roadmap from biomarker identification to potential regulatory approval and market introduction of a novel gout treatment (Fig. S1).

## Figures and Tables

**Table 1.** Identification details of biomarker strain *B. longum* through 16S sequencing and blast result from NCBI database

NCBI Reference	Organism	Length	Score	Identities
NR_145535.1	*Bifidobacterium longum* subsp. *suillum* strain Su 851	1428	2597 bits (1406)	1421/1428 (99%)
NR_117506.1	*Bifidobacterium longum* strain KCTC 3128	1488	2669 bits (1445)	1454/1458 (99%)
NR_043437.1	*Bifidobacterium longum* subsp. *infantis* strain ATCC 15697	1453	2662 bits (1441)	1450/1454 (99%)
NR_044691.2	*Bifidobacterium longum* subsp. *suis* strain ATCC 27533	1344	2460 bits (1332)	1341/1345 (99%)
NR_044693.2	*Bifidobacterium breve* DSM 20213	1513	2361 bits (1278)	1408/1470 (96%)
NR_040783.1	*Bifidobacterium cebidarum* strain CCUG 73785	1520	2361 bits (1278)	1409/1472 (96%)
NR_029137.1	*Bifidobacterium saeculare *strain RA161	1514	2361 bits (1278)	1397/1455 (96%)
NR_036856.1	*Bifidobacterium pullorum *strain B 145	1517	2357 bits (1276)	1397/1457 (96%)
NR_036855.1	*Bifidobacterium gallinarum *strain Ch206-5	1509	2340 bits (1267)	1395/1459 (96%)
NR_037117.1	*Bifidobacterium pseudocatenulatum *strain B1279	1513	2331 bits (1262)	1395/1459 (96%)
NR_037115.2	*Bifidobacterium dentium *strain B764	1518	2329 bits (1261)	1398/1463 (96%)

NCBI, National Center for Biotechnology Information

## Supplementary Information

Table S1. List of bacteria isolated from stool samples of healthy controls

Table S2. Identification of lactic acid bacterial strains based on 16S rRNA gene sequencing

Table S3. List of Primers that have been used for determining gene expression of Mice kidney RNA

Fig. S1. Comprehensive Reverse Translational Research Roadmap for Developing the Microbiome Therapeutic PMC72 as a Gout Treatment. We analyzed gout patients to isolate the biomarker strain PMC72 and conducted reverse translational studies for gout treatment in a potassium oxonate-induced mouse model. Our methodical plan consists of the following steps: (1) Biomarker identification through the collection of gut samples and subsequent metagenomic analysis, (2) Preclinical studies to evaluate the efficacy and safety of PMC72 in both in vivo and in vitro models, (3) Clinical trials encompassing Phases I, II, and III to assess therapeutic efficacy and safety in humans, and (4) Review and authorization process for regulatory approval followed by post-release monitoring, all aimed at the development of a novel gout treatment.

Fig. S2. Sample Sizes and Species Richness Comparison of 16s rRNA Gene Sequencing Across Different Treatment Groups: This figure shows species richness across different treatment groups as a function of sequence sample size. Each treatment group shows different patterns of species richness accumulation, with varying thresholds of sequence sample size. Data points represent individual sample measurements for each treatment group, and the dotted lines reflect the variability within each group.
