# Supplementary material for: Microbiome therapeutic PMC72 through reverse translational research in gout
Source: J Microbiol. 2025 May 27;63(5):e2501002. doi: 10.71150/jm.2501002 (PMC13577118; doi:10.71150/jm.2501002)
Supplement: Supplementary file 1 [file jm-2501002-Supplementary-Fig-S1.pdf]

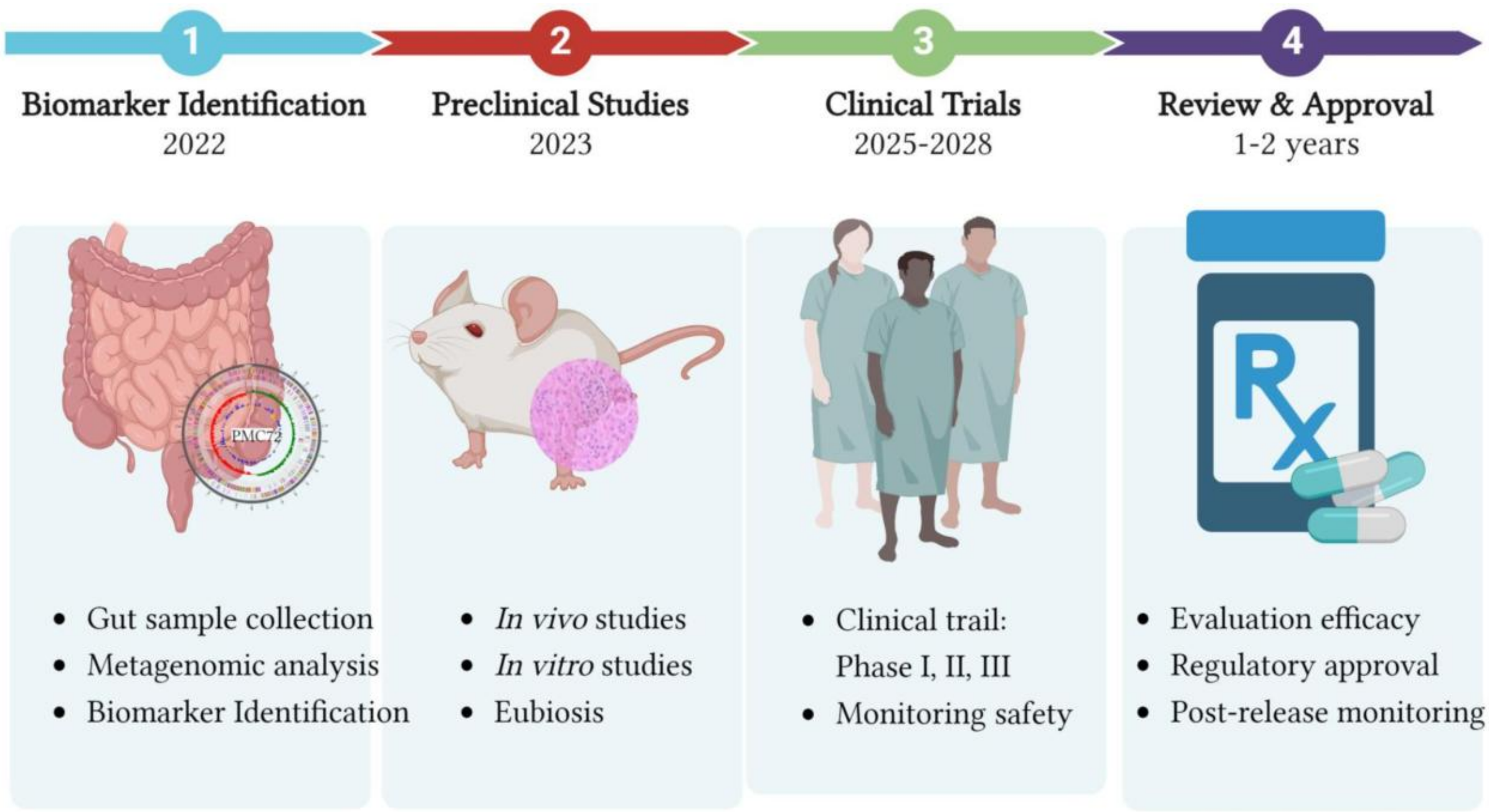

Fig. S1. Comprehensive Reverse Translational Research Roadmap for Developing the Microbiome Therapeutic PMC72 as a Gout Treatment. We analyzed gout patients to isolate the biomarker strain PMC72 and conducted reverse translational studies for gout treatment in a potassium oxonate-induced mouse model. Our methodical plan consists of the following steps: (1) Biomarker identification through the collection of gut samples and subsequent metagenomic analysis, (2) Preclinical studies to evaluate the efficacy and safety of PMC72 in both in vivo and in vitro models, (3) Clinical trials encompassing Phases I, II, and III to assess therapeutic efficacy and safety in humans, and (4) Review and authorization process for regulatory approval followed by post-release monitoring, all aimed at the development of ~~a~~ novel gout treatment.
