# Supplementary material for: Microbiome therapeutic PMC72 through reverse translational research in gout
Source: J Microbiol. 2025 May 27;63(5):e2501002. doi: 10.71150/jm.2501002 (PMC13577118; doi:10.71150/jm.2501002)
Supplement: Supplementary file 2 [file jm-2501002-Supplementary-Fig-S2.pdf]

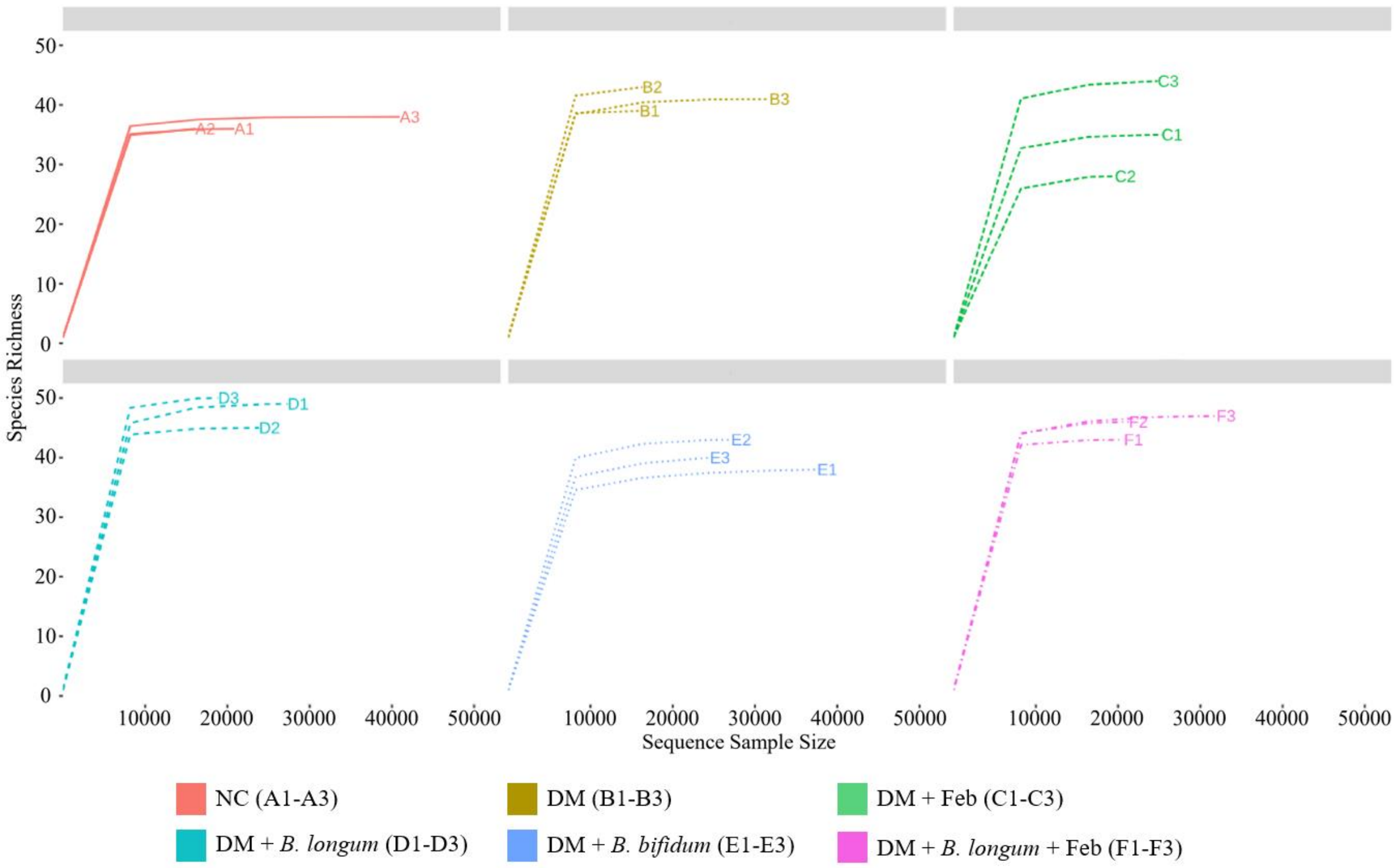

Fig. S2. Sample Sizes and Species Richness Comparison of 16s rRNA Gene Sequencing Across Different Treatment Groups: This figure shows species richness across different treatment groups as a function of sequence sample size. Each treatment group shows different patterns of species richness accumulation, with varying thresholds of sequence sample size. Data points represent individual sample measurements for each treatment group, and the dotted lines reflect the variability within each group.
