# Supplementary material for: Microbiome therapeutic PMC72 through reverse translational research in gout
Source: J Microbiol. 2025 May 27;63(5):e2501002. doi: 10.71150/jm.2501002 (PMC13577118; doi:10.71150/jm.2501002)
Supplement: Supplementary file 3 [file jm-2501002-Supplementary-Table-S1.pdf]

Table S1. List of bacteria isolated from stool samples of healthy controls

Culture condition	Stool sample IDs															
	N1	N2	N3	N4	N5	N6	N7	N9	N10	N11	N12	N13	N14	N15	N16	N17
BS agar, Mupirocin, Acetic acid, 37°C, Anaerobic	s1, s2, s4, s5	s8, s9, s13	s15, s17	s24, s25, s27	s31, s32	s35	s41, s42, s44, s45, s46	s52, 53	s54, s55	s60,	s62, s63	s67, s68	s70,	s73,	s79	s85, s86
BSM agar, BSM supplement, 37°C, Anaerobic	s6, s7	-	s18, s19	s28, s30	-	s38, s39, s40	s47, s48	-	s56, s57	-	s64, s65, s66	-	s71	s74, s75	s80, s81, s82, s83, s84	s87

s#: Represents individual stool sample IDs

-: Indicates no isolates detected in this condition

Comma (,): Used to separate individual sample IDs
