# Supplementary material for: Microbiome therapeutic PMC72 through reverse translational research in gout
Source: J Microbiol. 2025 May 27;63(5):e2501002. doi: 10.71150/jm.2501002 (PMC13577118; doi:10.71150/jm.2501002)
Supplement: Supplementary file 4 [file jm-2501002-Supplementary-Table-S2.pdf]

Table S2. Identification of lactic acid bacterial strains based on 16S rRNA gene sequencing

ID	NCBI Ref.	Organism	Scores (bits)	Identity
s81	NR_074802.2	*Bifidobacterium adolescentis* ATCC 15703	2359 (1277)	99%
	NR_118589.1	*Bifidobacterium adolescentis* JCM 15918	2333 (1233)	99%
s64	NR_040867.1	*Bifidobacterium animalis* subsp. *lactis* strain YIT 4121	2721 (1473)	99%
	NR_040867.1	*Bifidobacterium animalis* strain JCM 1190	2739 (1483)	99%
s42	NR_040867.1	*Bifidobacterium animalis* subsp. *lactis* strain YIT 4121	2730 (1478)	100%
	NR_180399.1	*Bifidobacterium castoris* strain LMG 30937	2492 (1349)	97%
s1	NR_040867.1	*Bifidobacterium animalis* strain JCM 1190	2739 (1483)	99%
	NR_040867.1	*Bifidobacterium animalis* subsp. *lactis* strain YIT 4121	2721 (1473)	99%
s15	NR_118793.1	*Bifidobacterium bifidum* strain KCTC 3202	2586 (1400)	99%
	NR_117505.1	*Bifidobacterium bifidum* strain KCTC 3202	2641 (1430)	99%
s6	NR_118793.1	*Bifidobacterium bifidum* strain KCTC 3202	2641 (1430)	99%
	NR_117764.1	*Bifidobacterium bifidum* ATCC 29521	2449 (1326)	99%
s65	NR_113873.1	*Bifidobacterium bifidum* strain NBRC 100015	2634 (1426)	99%
	NR_117505.1	*Bifidobacterium bifidum* strain KCTC 3202	2641 (1430)	99%
s18	NR_118793.1	*Bifidobacterium bifidum* strain KCTC 3202	2671 (1446)	99%
	NR_044771.1	*Bifidobacterium bifidum* strain KCTC 3202	2671 (1446)	99%
s57	NR_117764.1	*Bifidobacterium bifidum* ATCC 29521	2444 (1323)	99%
	NR_117505.1	*Bifidobacterium bifidum* strain KCTC 3202	2671 (1428)	99%
s30	NR_040783.1	*Bifidobacterium breve* DSM 20213	2656 (1438)	99%
	NR_043437.1	*Bifidobacterium longum* subsp. *infantis* strain ATCC 15697	2483 (1344)	97%
s9	NR_133982.1	*Bifidobacterium faecale* strain CU3-7	2337 (1265)	99%
	NR_074802.2	*Bifidobacterium adolescentis* ATCC 15703	2359 (1277)	99%
s87	NR_145535.1	*Bifidobacterium longum* subsp. *suillum* strain Su 851	2556 (1384)	99%
	NR_044691.2	*Bifidobacterium longum* strain ATCC 15707	2231 (1208)	98%
s85	NR_117506.1	*Bifidobacterium longum* strain KCTC 3128	2279 (1234)	99%
	NR_043437.1	*Bifidobacterium longum* subsp. *infantis* strain ATCC 15697	2254 (1220)	99%
s13	NR_117506.1	*Bifidobacterium longum* strain KCTC 3128	2163 (1171)	99%
	NR_145535.1	*Bifidobacterium longum* subsp. *suillum* strain Su 851	2276 (1232)	99%

s8	NR_145535.1	*Bifidobacterium longum* subsp. *suillum* strain Su 851	2597 (1406)	99%
	NR_044691.2	*Bifidobacterium longum* strain ATCC 15707	2121 (1148)	98%
s44	NR_145535.1	*Bifidobacterium longum* subsp. *suillum* strain Su 851	2639 (1429)	99%
	NR_043437.1	*Bifidobacterium longum* subsp. *infantis* strain ATCC 15697	2141 (1159)	99%
s71	NR_037117.1	*Bifidobacterium pseudocatenulatum* strain B1279	2529 (1369)	99%
	NR_041875.1	*Bifidobacterium catenulatum* strain DSM 16992	2508 (1358)	99%
