# Supplementary material for: Microbiome therapeutic PMC72 through reverse translational research in gout
Source: J Microbiol. 2025 May 27;63(5):e2501002. doi: 10.71150/jm.2501002 (PMC13577118; doi:10.71150/jm.2501002)
Supplement: Supplementary file 5 [file jm-2501002-Supplementary-Table-S3.pdf]

Table S3. List of Primers that have been used for determining gene expression of Mice kidney RNA

**Primers**	**Forward sequence**	**Reverse sequence**
NLRP3	5′-GTGGTGACCCTCTGTGAGGT-3′	5′-TCTTCCTGGAGCGCTTCTAA-3′
Caspase1	5′-TATCCAGGAGGGAATATGTG-3′	5′-ACAACACCACTCCTTGTTTC-3′
IL-1β	5′-GCCCATCCTCTGTGACTCA-3′	5′-AGTTGTCTGATTCCAGGTCTCCAT-3′
URAT1	5′-CGTGGGACCTGGTATGTAACT-3′	5′-CCAAACCTATCTGAGGCATGG-3′
GLUT9	5′-TTGCTTTAGCTTCCCTGATGTG-3′	5′-GAGAGGTTGTACCCGTAGAGG-3′
XOD	5′-ATGACGAGGACAACGGTAGAT-3′	5′-TCATACTTGGAGATCATCACGGT-3′
ß-Actin	5′-GGTCATCACTATTGGCAACG-3′	5′-ACGGATGTCAACGTCACACT-3′
